# Superheating of grain boundaries within bulk colloidal crystals

**DOI:** 10.1038/s41467-022-29254-z

**Published:** 2022-03-24

**Authors:** Xiuming Xiao, Lilin Wang, Zhijun Wang, Ziren Wang

**Affiliations:** 1 Key Laboratory of Soft Condensed Matter Physics and Smart Materials, College of Physics, Chongqing University, Chongqing, 401331 China; 2 State Key Laboratory of Solidification Processing, Northwestern Polytechnical University, Xi’an, 710072 China

**Keywords:** Surfaces, interfaces and thin films, Colloids

## Abstract

Whether grain boundaries (GBs) premelt is a longstanding question, because of the difficulty of direct experimental tests. Here, we focused an optical beam to locally heat single GBs within bulk hard-sphere colloidal crystals, observing the melting dynamics at single-particle resolution by video microscopy. The melting point is determined by analysing both the Lindemann parameter and the critical nucleus size for homogeneous nucleation. We found that all the GBs, including the high-energy GBs, can be superheated and melt via a heterogeneous nucleation mechanism. Based on the classical nucleation theory of GBs, we measured the incubation time and contact angle of the critical nucleus to compute all relevant kinetic factors, as well as the energy barrier, nucleation rate and the diffusion coefficient at the solid–liquid interface under weak superheating. The superheat limits of GBs with various misorientations have also been measured to further explore the instability mechanism. Under traditional uniform heating, premelting occurs only at triple junctions, whereas GBs retain their original structures up to the melting point. The premelted regions at triple junctions further interrupt high-energy GBs from superheating, through intrusion by uniform liquid layers. Overall, our experiments confirm the existence of superheating of GBs.

## Abstract (web summary)

Understanding the dynamics of grain boundaries and their melting behaviour is important for controlling the mechanical properties of materials. Now, experiments show that grain boundaries can be superheated, and that they melt via a nucleation mechanism.

## Introduction

Grain boundaries (GBs) dominate mechanical properties and exert decisive impacts on microstructures of materials^1,2^. Therefore, the structural stability of GBs at elevated temperature constitutes a fundamental concern in materials science and condensed matter physics^3,4^, because GBs can trigger heterogeneous melting and thus alter material properties. The possibility that GBs would have liquid layers at the melting point was first discussed by Gibbs^5^. Since then, the melting behaviours of GBs have been extensively discussed by theoretical models^6–11^ and simulations^2,10,12–28^ in the characterisation of various ceramic, metallic and icy materials. However, the question of whether GBs melt below the bulk melting temperature *T*_m_ (i.e., premelting of GBs) or can stay above it as a metastable superheating state remains inconclusive, due to the inherent difficulty in directly validating such theoretical and computational models with experimental measurements, which are hidden within the bulk of three-dimensional (3D) materials^3,29–31^. Therefore, almost all relevant experiments have reported indirect evidence on the melting of GBs around *T*_m_^31–41^.

Colloids are outstanding model systems for visualising this melting process because the dynamics of each colloidal particle can be directly tracked by optical video microscopy. Alsayed et al.^29^ reported the first direct evidence that melting starts by “premelting”^30^ at defects, particularly at GBs, within bulk colloidal crystals composed of thermosensitive microgel *N*-isopropylacrylamide (NIPA) spheres.

However, the lack of systematic investigations of GBs with different misorientations and the challenge of accurately determining the bulk melting point still make it difficult to reveal the nature of GBs melting. Furthermore, aside from the influence from the substrates or surfaces that favour wetting, preexisting defects within the crystals can also affect each other, resulting in a complex melting process. Consequently, it is necessary to extract the melting behaviour for single GBs.

**Fig. 1:**
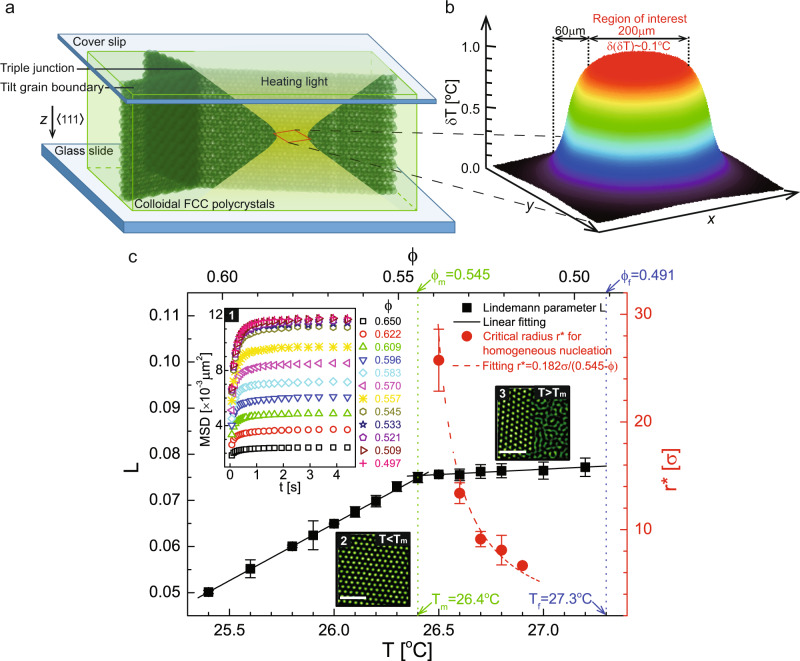
The optical heating and determination of the melting point. **a** The schematic of optical heating. **b** The measured temperature profile on the object plane. **c** Determination of the bulk melting point. Squares: Lindemann parameter *L* as a function of temperature (and volume fraction *ϕ*). Circles: the critical radii for homogeneous nucleation inside a superheated single crystalline domain. The vertical dotted lines denote the melting and freezing points respectively. Inset 1: 2D mean-square displacement (MSD) which saturates after 4 s. *L* is computed from the MSD plateau value. Insets 2 and 3: cross-sections of real images of the crystals and the bulk solid–liquid coexistence before and after melting, respectively. Error bars correspond to the standard deviation. Scale bars: 5 μm.

In this study, to minimise the interference arising among various defects, we focused a beam of light to locally heat single GBs as well as other types of single defects within the NIPA colloidal crystals, and investigated the corresponding melting process by video microscopy. This local heating technique was initially developed to investigate homogeneous melting^42,43^. Meanwhile, we accurately located the melting point through both monitoring a sudden slope change in the Lindemann parameters and extrapolating the critical radii of homogeneous nucleation to infinity (Fig. 1c). We found that all the GBs can be superheated and melt undergoing a nucleation mechanism.

## Results

### The experimental system

We synthesised the NIPA spheres referring to the methods described in ref. ^44^. The diameter of the spheres *σ* changes almost linearly from 1.14 μm at 25.5 °C to 1.06 μm at 27.5 °C (Supplementary Fig. 1). The measured pair interaction (Supplementary Fig. 2) is short-range repulsive that is close to hard spheres. We prepared the sample by loading the NIPA colloidal suspension into an 18.0 × 3.0 × 0.2 mm^3^ glass channel and annealed it into a face-centred cubic (fcc) polycrystal with a few large domains in several days. By tuning the temperature, we precisely changed the volume fraction over a substantial range to melt and recrystallise the sample.

We adjusted the ambient temperature *T*_ambient_ by uniformly heating ~1.8 cm^2^ of the sample with temperature controllers (Bioptechs) at a resolution of 0.1 °C. Besides the uniform heating method, to extract the melting behaviours of single GBs, we focused a beam of light emitted from a 100 W mercury lamp by the objective through the reflection mode to heat single GBs in the interior of the polycrystal (Fig. 1a), which gives rise to an extra temperature increase *δT* ≈ 1.0 °C in the heated region (Fig. 1b). Typically, the maximum temperature (*T*_ambient_ + *δT*) at the centre of the object plane decays by 0.1 °C (i.e., 0.5% in volume fraction) in the 4*π*/3(100 μm)^2^ (i.e., approximately the scale of field of view) (Fig. 1b) by 60 μm (±70 layers in the *z* direction) region of interest. The temperature in this region, which contains ~2.0 × 10^6^ particles, is sufficiently uniform, as indicated by random nucleation on the GB interface. While we performed heating, we observed the sample in the transmission mode of the same microscope. More experimental details are available in Methods.

### Determination of the melting point

The basic way in determining the melting point is to find the state at which the Gibbs free energy per atom of the solid and liquid phases are equal. However, it is hard to accurately calculate the Gibbs free energy in colloidal systems. Herein, we adopted the Lindemann parameter *L* as a benchmark to determine the melting point. Lindemann parameter *L*^45^ is a measurement of vibrating amplitude for crystalline particles relative to the equilibrium position. It can be calculated as (Supplementary Note 1):

1 $$L=\frac{1}{{r}_{{{{{{{{\rm{nn}}}}}}}}}}\sqrt{\frac{3}{4}\langle {{{{{{{\rm{[}}}}}}}}{{{{{{{\bf{r}}}}}}}}(t\to \infty )-{{{{{{{\bf{r}}}}}}}}(0){{{{{{{{\rm{]}}}}}}}}}^{2}\rangle },$$

where *r*_nn_ is the crystal nearest-neighbour distance and 〈[**r**(*t* → *∞*) − **r**(0)]^2^〉 is the asymptotic value of the particles’ two-dimensional mean-square displacement (MSD). The MSD reaches a plateau due to the caging by nearest neighbours in 4 s (inset 1 of Fig. 1c).

When we uniformly heated the crystals (inset 2 of Fig. 1c), *L* linearly increases with *T* and becomes almost a constant when the system crosses the melting line reaching the equilibrium bulk solid–liquid coexistence regime (inset 3 of Fig. 1c), due to a constant volume fraction of bulk crystals in the regime, which causes a turning in the slope. The fitted *T*_m_ = 26.4 °C (more precisely 26.415 °C) with *L* = 0.075 (solid lines in Fig. 1c).

Before locally heating a single GB, we first casted the heating light inside the bulk of a large perfect crystalline domain that is partly edged with the GB. Then we searched for the melting point by increasing *T*_ambient_ until *L* = 0.075. Unlike the case with uniform heating, *L* could exceed 0.075 when the degree of superheating Δ*T* ≡ *T*_ambient_ + *δT* − *T*_m_ ≡ *T* − *T*_m_ > 0 (i.e., Δ*ϕ* ≡ *ϕ*_m_ − *ϕ* > 0), corresponding to a superheated crystal.

To exclude the possibility that the bulk solid–liquid coexistence (inset 3 of Fig. 1c) is a pseudo equilibrium state caused by GB or triple junction premelting, we measured the critical radius r^*^ (more details in Methods) for homogeneous nucleation at superheating (circles in Fig. 1c) to verify that the measured *T*_m_ is indeed the bulk melting point. According to the classical nucleation theory, r^*^ is inversely proportional to Δ*ϕ* under weak superheating when Δ*ϕ* ≲ 0.025^42,43^. Our measured r^*^ = 0.182*σ*/(0.545 − *ϕ*). The fitted parameter 0.182 is close to the theoretical number 0.195 (Supplementary Note 2), confirming that 26.4 °C is the melting point. Notably, when r^*^ ≳ 25*σ* (Fig. 1c), the corresponding temperature is already within the window of 0.1 °C from *T*_m_, so one data point is sufficiently accurate to locate the melting point. Our method to determine the melting point is self-consistent and does not rely on the known phase diagram. This method can also apply to attractive colloidal systems^46^.

### Premelting at triple junctions

**Fig. 2:**
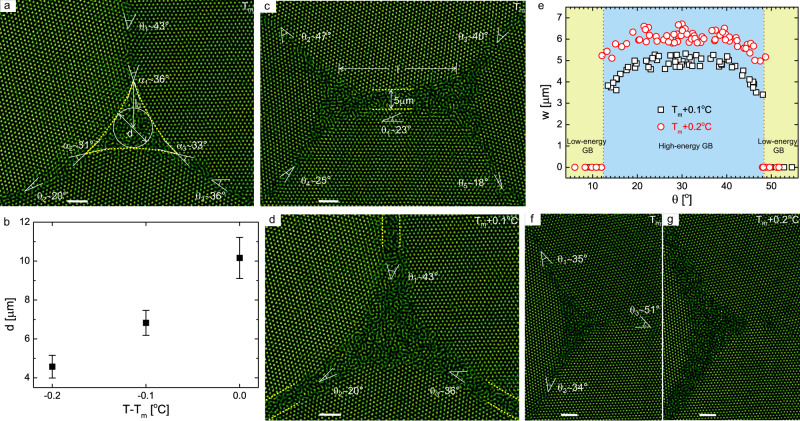
Premelting at triple junctions. **a** Real image of a typical premelted triple junction at the melting point. *l*_0_ is the distance between the centre of the inner tangent circle of the premelted pocket and a crystal–crystal–liquid triple junction. **b** The size of the premelted pocket *d* increases as *T* approaches *T*_m_. **c** Two close triple junctions help wet the GB in between and cause pseudo “premelting”. **d** The GBs melt into liquid layers via intrusion once *T* > *T*_m_. **e** The equilibrium widths of the melted GBs *w* as a function of misorientation *θ* at *T*_m_ + 0.1(2) °C. The inclination angles are arbitrarily chosen under each *θ*. **f**, **g** Melting behaviour in the case of a low-angle GB connected to the triple junction. It is noteworthy that both uniform and local heating methods yield the same **a**–**g**, which means that the glass walls in our system have a minor effect on melting behaviour of triple junctions. Since the cross-section of the liquid region is uniform along the *z* direction, we fixed the object plane in the middle range of the glass channel. Error bars correspond to the standard deviation. Scale bars: 5 μm.

**Fig. 3:**
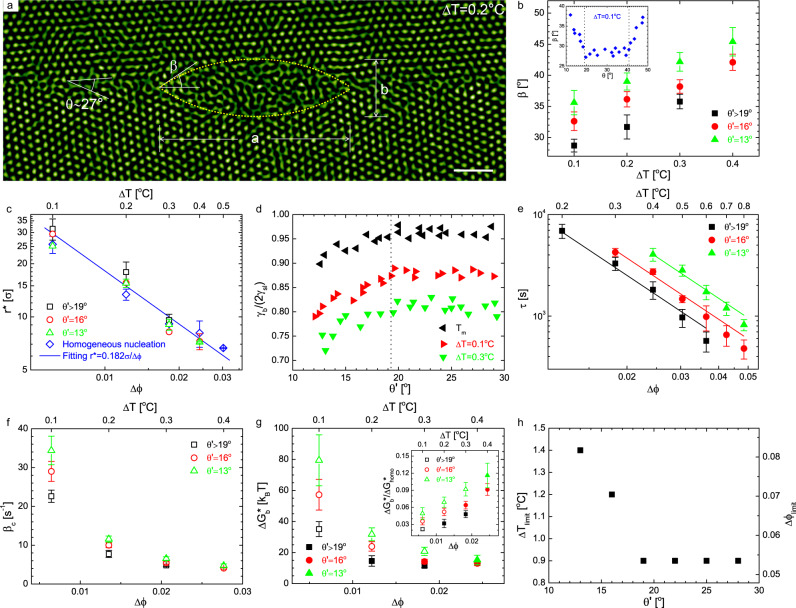
Superheating of GBs. **a** Largest cross-section of the critical nucleus along a high-energy GB with *θ* = 27°. **b** Variation of the contact angle *β* with ${\theta }^{\prime}$ under different degrees of superheating. ${\theta }^{\prime}\equiv \min \{\theta ,6{0}^{\circ }-\theta \}$. Inset: symmetrical distribution of *β* at *θ* = 30°. **c** The measured critical radii r^*^ for homogeneous nucleation and heterogeneous nucleation on GBs, fitted by (Δ*ϕ*)^−1^ (solid line). **d** The measured ratio *γ*_b_/(2*γ*_sl_) depending on ${\theta }^{\prime}$. **e**, **f** The incubation time *τ* and transport rate of particles adding to the critical nucleus *β*_c_, as a function of the degree of superheating for ${\theta }^{\prime}=1{3}^{\circ }$ (triangles), 16° (circles), >19° (squares). *τ*s are fitted by (Δ*ϕ*)^−2^ (solid lines). **g** The nucleation energy barriers ${{\Delta }}{G}_{{{{{{{{\rm{b}}}}}}}}}^{* }$ and ${{\Delta }}{G}_{{{{{{{{\rm{b}}}}}}}}}^{* }/{{\Delta }}{G}_{{{{{{{{\rm{homo}}}}}}}}}^{* }$ (inset) computed based on the classical nucleation theory. Solid symbols denote the energy barriers under which the incubation times are measured in **e**, otherwise the data points are plotted with open symbols. **h** The measured superheat limits variation with ${\theta }^{\prime}$. All the measurements under each ${\theta }^{\prime}$ are obtained by averaging GBs over different inclination angles. Error bars correspond to the standard deviation. Scale bar: 5 μm.

When uniformly heating the sample, triple junctions start to premelt at *T*_m_ − 0.2 °C. By contrast, all the GBs keep their original structures until *T*_m_ (Fig. 2a, c, f and Supplementary Video 1), including rough GBs with significant curvature. We measured a nonzero dihedral angle *α* at *T*_m_ (Fig. 2a), again suggesting that the liquids only partially wet the GBs. Note that within one or two layers around the GB interface (Figs. 2a and 3a and Supplementary Video 1), the structure is intrinsically disordered^29,47,48^, leading to some blur in the image. The blur does not expand in images captured below *T*_m_, in contrast to the successive growth of the premelted region at the triple junction as the temperature rises (Fig. 2b and Supplementary Video 1). In the simplest scenario, ignoring the triple line energy, melting should occur first at triple junctions because the wetting criterion for triple junctions ($\sqrt{3}{\gamma }_{{{{{{{{\rm{sl}}}}}}}}}\le {\gamma }_{{{{{{{{\rm{b}}}}}}}}}$)^32,49^ is weaker than that for GBs (2*γ*_sl_ ≤ *γ*_b_), as observed in aluminium^31^ and fcc ^4^He^35,36^ systems. Here, *γ*_sl_ and *γ*_b_ denote the interfacial tensions at the solid–liquid interface and at the GB, respectively. The pocket size *d* of a premelted triple junction, defined as the diameter of the inner tangent circle constrained by the three solid–liquid interfaces^31,49^, increases continuously as *T* rises and remains finite at the melting point (Fig. 2a, b and Supplementary Video 1).

However, two close triple junctions can cause pseudo “premelting” of the GB in between at *T* < *T*_m_ when their distance is smaller than a critical value. The critical distance mainly depends on *θ*_1_ (Fig. 2c) and increases as the temperature approaches the melting point. As shown in Fig. 2c, we measured the critical distance under which the width of wetting layers *w* is 5 μm (i.e., the width for *θ*_1_ = 23° at *T*_m_ + 0.1 °C in Fig. 2e). Basically, the critical distance *l* = 2*l*_0_ + *δl* (*δl* ≈ 10 layers). Ten layers arise because four to five neighbouring layers of crystalline lattices from the solid–liquid interface vibrate more strongly than bulk ones^43^. *l* governs the quantification of the effect of grain size on GB melting behaviour: the grain size must be larger than the order of scale *l*^3^ (~2.0 × 10^4^*σ*^3^) to ensure a dry GB at the melting point. In other words, the shear modulus should drop as the grain size decreases and vanish when the size is smaller than 2.0 × 10^4^.

Once *T* rises above *T*_m_, GBs are quickly intruded by uniform liquid layers that bridge each premelted triple junction (Fig. 2d and Supplementary Video 1), then each uniform layer simply widens as *T* increases (Fig. 2e and Supplementary Video 1). At *T*_m_ + 0.1 °C, the equilibrium width *w* of the melting layer gradually increases with the misorientation *θ* and becomes a constant when 19° < *θ* < 41° (Fig. 2e), displaying a similar profile as *γ*_b_ variation with *θ* (inset of Fig. 3b). This similarity suggests that the release of interfacial energy *γ*_b_ destabilises the crystalline phase, facilitating GB melting. From the thermodynamic perspective, to proceed this transition, the free energy change must be negative, which leads to *w* ≥ (2*γ*_sl_ − *γ*_b_)/(*ρ*_l_Δ*μ*), where Δ*μ* represents the chemical potential difference between solids and liquids, and *ρ*_l_ is the number density of the liquid phase (Supplementary Note 2). This gives the estimated *w* ≥ 3.6 μm at Δ*T* = 0.1 °C (assuming Δ*μ* unchanged during this transition), in line with our measurement of 5 μm (Fig. 2e). From the dynamic perspective, it would be interesting to explore the time evolution of *w* under different conditions.

Figure 2a–d presents the case in which three high-energy GBs are connected to a triple junction. If any one of the GBs is in relatively low energy (*θ* < 12° or *θ* > 48°) (Fig. 2e), as described in terms of individual dislocations separated by distances greater than a few lattice spacing, we still observed premelting at the triple junction (Fig. 2f, g), but the wetting region at the triple junction cannot penetrate the low-energy GB (Fig. 2g) at *T* > *T*_m_.

### Superheating of GBs

In contrast to the intrusion melting of GBs from the premelted regions at triple junctions under uniform heating (Fig. 2d), when we locally heated a single GB, it displayed a heterogeneous nucleation mechanism through that subcritical nuclei formed and disappeared during the incubation period until one of them reached the critical size (Fig. 3a and Supplementary Video 2). Nucleation was observed for GBs with any misorientation, which is a hallmark to prove that GBs can be superheated, agreeing with the fact that we did not observe the premelting of GBs at *T* ≤ *T*_m_ (Fig. 2a, c, f and Supplementary Video 1).

In the simplest premelting theory for GBs^3,7,8,50^, a premelted GB is represented as a uniform liquid layer at width *w* between two sharp solid–liquid interfaces with the interaction described by a disjoining potential V(*w*)^8,26^. The change of the Gibbs free energy per unit of GB area is taken as Δ*G*(*w*) = −*w*Δ*μρ*_l_ + (2*γ*_sl_ − *γ*_b_) + V(*w*). V(*w*) has the generic form ${{{{{{{\rm{V}}}}}}}}(w)=({\gamma }_{{{{{{{{\rm{b}}}}}}}}}-2{\gamma }_{{{{{{{{\rm{sl}}}}}}}}})[{C}_{{{{{{{{\rm{1}}}}}}}}}\exp (-w/{\delta }_{{{{{{{{\rm{1}}}}}}}}})-{C}_{{{{{{{{\rm{2}}}}}}}}}\exp (-w/{\delta }_{{{{{{{{\rm{2}}}}}}}}})]$ with *C*_1_ − *C*_2_ = 1. The two exponential terms respectively quantify the contributions of the short-range and long-range parts of the interaction with decay lengths *δ*_1_ < *δ*_2_. Practically, V(*w*) is not derived directly from a microscopic theory, instead V(*w*) is chosen to interpret different scenarios of GB melting. The equilibrium value of *w* is the one corresponding to a minimum in the free energy. Since GBs remain dry up to the melting point in our experiments, the interaction between two interfaces is attractive for small *w*, which means $(\partial {{\Delta }}G(w)/\partial w){| }_{{T}_{{{{{{{{\rm{m}}}}}}}}},w = 0}\approx (2{\gamma }_{{{{{{{{\rm{sl}}}}}}}}}-{\gamma }_{{{{{{{{\rm{b}}}}}}}}}){C}_{{{{{{{{\rm{1}}}}}}}}}\, > \,0$. If 2*γ*_sl_ − *γ*_b_ < 0, GBs will become liquid layers by crossing an energy barrier. In actuality, the energy barrier does not exist because of the premelted triple junctions. Therefore, 2*γ*_sl_ − *γ*_b_ > 0, in line with our observation of the nucleation mechanism at superheating that requires 2*γ*_sl_ > *γ*_b_ (i.e., a finite free energy barrier), and direct measurements in Fig. 3d.

Figure 3a shows the critical nucleus at *θ* = 27° when Δ*T* = 0.2 °C (see Supplementary Video 3 for the 3D scan). According to the 3D scan (Supplementary Video 3), the critical nuclei are roughly composed of two symmetrical abutted spherical caps on either side of the GB face judged by the measured equal scale lengths along the largest cross-section (i.e., *a* as labelled in Fig. 3a) and along the *z* direction, agreeing with the assumption of the classical nucleation theory^51^. Apart from the nucleus, the rest part of the GB remains dry (Fig. 3a) as observed below *T*_m_ (Fig. 2a).

The contact angle *β* (Fig. 3b), as sketched in Fig. 3a, is measured by ${\cos }^{-1}[({a}^{2}-{b}^{2})/({a}^{2}+{b}^{2})]$. For a given Δ*T*, *β* basically reflects the magnitude of *γ*_b_ (Fig. 3d). *β* reaches the bottom of the basin (inset of Fig. 3b) when 19° < *θ* < 41°. Considering the nearly symmetrical distribution of *γ*_b_ at *θ* = 30° (Fig. 2e and inset of Fig. 3b), we introduce another equivalent misorientation angle ${\theta }^{\prime}\equiv \min \{\theta ,6{0}^{\circ }-\theta \}$ in subsequent quantitative measurements to indicate the magnitude of *γ*_b_. In addition, we just look into three representative angles ${\theta }^{\prime}=1{3}^{\circ },1{6}^{\circ }, > 1{9}^{\circ }$ for simplicity. As shown in Fig. 3b, *β* slightly increases with Δ*T*. The small error bar of *β* for each ${\theta }^{\prime}$ guarantees that the inclination angle has a minor effect on *γ*_b_ relative to the misorientation. When Δ*T* ≥ 0.5 °C (i.e., just above the weak superheating regime), the small critical nuclei are vulnerable to thermal fluctuations and begin to deviate from a spherical cap shape.

The measured critical radii ${r}^{* }=b/[2(1-\cos \beta )]$ collapse into a single curve (Fig. 3c), including the data obtained for homogeneous nucleation (circles in Fig. 1c), which suggests the critical radii along the GBs depend little on *θ* or ${\theta }^{\prime}$ (i.e., *γ*_b_), as predicted by the classical nucleation theory^51^ (Supplementary Note 3). In fact, heterogeneous nucleation on GBs is analogous to homogeneous nucleation on GB planes.

According to Antonow’s rule^52^, ${\gamma }_{{{{{{{{\rm{b}}}}}}}}}/(2{\gamma }_{{{{{{{{\rm{sl}}}}}}}}})=\cos \beta$, maintaining the balance of interfacial tensions. As Fig. 3d shows, at a given Δ*T*, *γ*_b_ increases with ${\theta }^{\prime}$ because of the increase in dislocation density attributed to lattice misfit, and saturates when ${\theta }^{\prime}$ exceeds 19°. At this stage, the GBs are totally incoherent. The *γ*_b_/(2*γ*_sl_) at the melting point is similarly obtained by measuring *α* (Fig. 2a). We note that *γ*_b_/(2*γ*_sl_) weakly increases as Δ*T* decreases. Near the melting point, *γ*_b_/(2*γ*_sl_) ≤ 0.95 for GBs with various misorientations. Given that r^*^ = 0.182*σ*/Δ*ϕ* (Figs. 1c and 3c) and Δ*μ*/*k*_B_*T* = 6.7Δ*ϕ* (Supplementary Note 2), we obtained *γ*_sl_ = 0.56*k*_B_*T*/*σ*^2^. Meanwhile, *γ*_b_ for ${\theta }^{\prime}\, > \,1{9}^{\circ }$ was directly measured to be 0.78*k*_B_*T*/*σ*^2^ near the melting point, through the way of quantifying the fluctuations of unperturbed GBs^53^ (Supplementary Note 4). Thus, *γ*_b_/(2*γ*_sl_) = 0.70, which strengthens the result of *γ*_b_/(2*γ*_sl_) < 1.

The incubation time *τ*, the amount of time required for the development of steady-state size distribution of subcritical nuclei, can be estimated by counting the average waiting time for the first postcritical nucleus to form after turning on the heating light. The measured *τ* (Fig. 3e) obeys *τ* ∝ Δ*ϕ*^−2^ for all the three ${\theta }^{\prime}$ under weak superheating, which agrees with the theoretical derivation (Supplementary Note 3).

Theoretically, the incubation time *τ* is derived as (Supplementary Note 3):

2 $$\tau =\frac{4{k}_{{{{{{{{\rm{B}}}}}}}}}T{\gamma }_{{{{{{{{\rm{sl}}}}}}}}}}{{{\Delta }}{\mu }^{2}{l}_{{{{{{{{\rm{a}}}}}}}}}{\rho }_{{{{{{{{\rm{l}}}}}}}}}{{\Gamma }}}(2+\cos \beta )(1-\cos \beta ),$$

where Γ is the rate of a successful jump for a particle crossing the nucleus interface. ${l}_{{{{{{{{\rm{a}}}}}}}}}\approx \sigma {\phi }_{{{{{{{{\rm{l}}}}}}}}}^{-1/3}$ is the particle spacing. The three curves in Fig. 3e yield a fitted Γ ≈ 0.02 s^−1^ (i.e., 0.06*D*_0_/*σ*^2^). The free-diffusion coefficient (*D*_0_ = 0.357*σ*^2^/s) is calculated by the Stokes-Einstein equation. Meanwhile, the computed effective diffusion coefficient at the solid–liquid interface ${D}_{{{{{{{{\rm{interface}}}}}}}}}={{\Gamma }}{l}_{{{{{{{{\rm{a}}}}}}}}}^{2}\approx 0.03\,\mu$m^2^/s (i.e., 0.07*D*_0_), which is closer to the long-time diffusion coefficient *D*_L_ ≈ 0.04*D*_0_, in comparison to the short-time diffusion coefficient *D*_S_ ≈ 0.188*D*_0_. Here, ${D}_{{{{{{{{\rm{L}}}}}}}}}={D}_{{{{{{{{\rm{0}}}}}}}}}{(1-{\phi }_{{{{{{{{\rm{l}}}}}}}}}/0.58)}^{1.74}$ and ${D}_{{{{{{{{\rm{S}}}}}}}}}={D}_{{{{{{{{\rm{0}}}}}}}}}{(1-{\phi }_{{{{{{{{\rm{l}}}}}}}}}/0.64)}^{1.17}$ for hard spheres^54^. *D*_interface_ is a useful variable in calculating the growth rates during crystallisation and melting^43^. It is conceivable that *D*_interface_ involves combining properties of *D*_L_ and *D*_S_.

Under stronger superheating (Δ*T* ≥ 0.5 °C), *τ* becomes smaller than expected (Fig. 3e). We attribute this deviation primarily to the curvature effect that considerably lowers *γ*_sl_ when the nucleus radius decreases to (3 − 6)*σ*^55,56^.

The steady-state nucleation regime follows the incubation regime. The nucleation rate $I=\kappa \exp (-{{\Delta }}{G}_{{{{{{{{\rm{b}}}}}}}}}^{* }/{k}_{{{{{{{{\rm{B}}}}}}}}}T)$, where the kinetic prefactor *κ* = *Zβ*_c_*ρ*_A_ and ${{\Delta }}{G}_{{{{{{{{\rm{b}}}}}}}}}^{* }$ is the height of the heterogeneous nucleation barrier. *Z* is the Zeldovich factor, *β*_c_ is the rate of particles adding to the critical nucleus, and *ρ*_A_ is the number of heterogeneous nucleation sites per unit area on GB planes. ${\beta }_{{{{{{{{\rm{c}}}}}}}}}={{\Gamma }}{S}_{{n}^{* }}$, where ${S}_{{n}^{* }}=4\pi {l}_{{{{{{{{\rm{a}}}}}}}}}{\rho }_{{{{{{{{\rm{l}}}}}}}}}{r}^{* 2}(1-\cos \beta )$ is the number of particles adjacent to the solid–liquid interface when the nucleus is at the critical size n*. Generally, as Fig. 3f shows, *β*_c_ decreases with increasing degree of superheating and *γ*_b_. The ratio ${{\Delta }}{G}_{{{{{{{{\rm{b}}}}}}}}}^{* }/{{\Delta }}{G}_{{{{{{{{\rm{homo}}}}}}}}}^{* }=[(2+\cos \beta ){(1-\cos \beta )}^{2}/2]$ (inset of Fig. 3g) (Supplementary Note 3), where ${{\Delta }}{G}_{{{{{{{{\rm{homo}}}}}}}}}^{* }$ represents the energy barrier for homogeneous nucleation. Larger ${\theta }^{\prime}$ leads to a smaller ${{\Delta }}{G}_{{{{{{{{\rm{b}}}}}}}}}^{* }$ at a given Δ*ϕ* (Fig. 3g), as expected. It is worth noting that when ${{\Delta }}{G}_{{{{{{{{\rm{b}}}}}}}}}^{* }\,\gtrsim\, 18\,{k}_{{{{{{{{\rm{B}}}}}}}}}T$, as indicated by the open symbols in Fig. 3g, the associated incubation time *τ* is not measurable within the duration of our experiments (~2.5 h). For the solid-symbol data in Fig. 3g, the corresponding calculated nucleation rates range from 10^−8^ to 10^−7^ μm^−2^ s^−1^ (i.e., 10^−8^−10^−7^*D*_0_/*σ*^4^). The kinetic prefactor *κ* ≈ 0.15*D*_0_/*σ*^4^ under weak superheating, where the Zeldovich factor $Z={[{{\Delta }}{G}_{{{{{{{{\rm{b}}}}}}}}}^{* }/(3\pi {k}_{B}T)]}^{1/2}/{n}^{* }$ ranges from 10^−4^ to 10^−3^ and *ρ*_A_ ≈ 1.5 μm^−2^, assuming a bilayer GB. Hopefully, all the results can help guide later simulations and experiments.

At the superheat limit, the energy barrier becomes comparable to *k*_B_*T* and any fluctuated liquid protrusion on the GB tends to grow. The GB transfers from a metastable state to an unstable state and melts from everywhere, presenting itself in a way of widening as a whole (Supplementary Video 4). Our measured superheat limits Δ*ϕ*_limit_ are respectively 0.082, 0.071, 0.054 (i.e., 15%, 13%, 10% above *ϕ*_m_) for ${\theta }^{\prime}=1{3}^{\circ },1{6}^{\circ }, > 1{9}^{\circ }$ (Fig. 3h). As ${\theta }^{\prime}$ decreases, Δ*ϕ*_limit_ increases, reaching 0.125 (~20% above *ϕ*_m_) for a perfect crystal. Prediction of superheat limit is beyond the scope of the classical nucleation theory. Hopefully, our experiments can help clarify the GB instability mechanism.

**Fig. 4:**
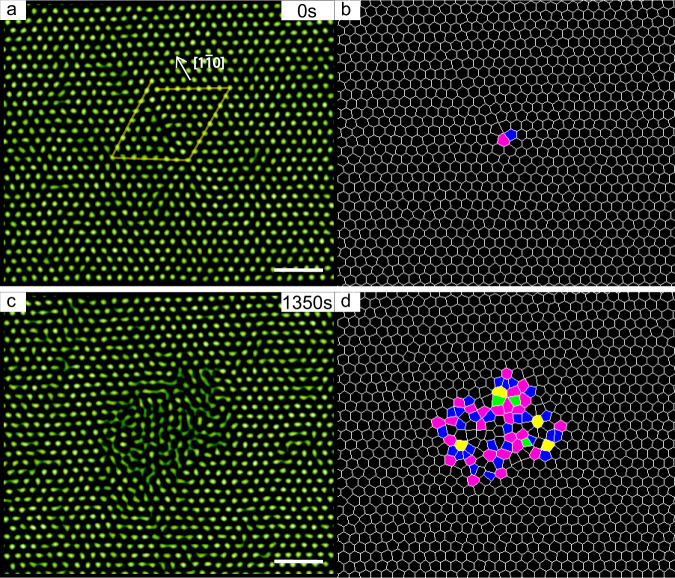
Nucleation on a single dislocation. **a** and **c** are real images, **b** and **d** are the corresponding Voronoi diagrams for better visualisation. **c** and **d** show the state 1350 s after turning on the heating light. The degree of superheating Δ*T* = 1.0 °C (i.e., Δ*ϕ* = 0.059). Voronoi cells are coloured according to the number of the nearest neighbours: 4-green; 5-blue; 6-black; 7-magenta; 8-yellow. The Burgers circuit in **a** reveals a full dislocation with the Burgers vector $[1\overline{1}0]/2$ of the lattice constant, which is featured as a 5–7 pair in the Voronoi cells. The nucleation has just started on the object plane. Scale bars: 5 μm.

Thus far, the discussion on GB nucleation has been restricted to high ${\theta }^{\prime}$. For ${\theta }^{\prime} < 1{2}^{\circ }$, we found that liquid pools nucleate around the dislocation cores. Furthermore, under the limit condition of ${\theta }^{\prime}\to {0}^{\circ }$, by locally heating isolated single dislocations, we found that single dislocations tend to attract momentarily appeared defects nearby and suppress the nucleation ~27 *σ* around their cores (Fig. 4 and Supplementary Video 5). Two times of 27 *σ* corresponds to the distance between neighbouring dislocations in GBs with ${\theta }^{\prime}\approx {1}^{\circ }$. So it can be speculated that when ${\theta }^{\prime}\lesssim {1}^{\circ }$, the strain fields between dislocations are too weak to obviously affect each other on the melting behaviour. Therefore, the situation can be simplified to the nucleation on single dislocations^57^. Note that during the nucleation on single dislocations (Fig. 4 and Supplementary Video 5), we did not observe sensible lattice distortions, indicating little change in the elastic strain energy, in contrast to the significant change in atomic systems.

## Discussion

Overall, we reported on a real-space study of GB melting within bulk colloidal crystals by video microscopy. The observed nucleation on single superheated GBs is a qualitative phenomenon to unambiguously determine the free-energy ratio *γ*_b_/(2*γ*_sl_) < 1, which means no premelting of GBs and GB wetting is a phase transition, more than an equilibrium phenomenon. Although at small grain size (≲10^4^), GBs could appear pseudo “premelting" due to adjacent premelted triple junctions, *γ*_b_/(2*γ*_sl_) < 1 still holds, which sets a constraint to the system and offers many other implications for the properties of polycrystalline materials.

Our conclusion of no premelting of GBs in hard-sphere systems agrees with some previous molecular dynamics (MD) simulations^17–20,23^. Meanwhile, MD simulations have also revealed many examples of extensive structural disordering below the bulk melting temperature. Melting scenarios of GBs differ depending on many circumstances, such as the GB bicrystallography and the particle interactions. Accordingly, disjoining potentials may be repulsive, attractive or a combination of both. Here, our experiments verify that the superheating of GBs exists.

## Methods

### Sample preparation

The thermosensitive microgel NIPA spheres are suspended in 10 mM acetic acid buffer solution. The nearly linear relationship between diameter and temperature *σ*(*T*) (Supplementary Fig. 1) was measured by analysing the image of the isolated particles that were stuck on the glass wall in a dilute suspension. This result is consistent with an alternative measurement by dynamic light scattering. The polydispersity of the particle sizes is calculated to be 3% based on the imaging processing.

We calibrated the particle diameter so that the melting volume fraction *ϕ*_m_ is 54.5% (dotted lines in Fig. 1c and Supplementary Fig. 1), the same as that of hard spheres. Subject to this definition, the freezing volume fraction *ϕ*_f_ is at 49.1% (dotted lines in Fig. 1c and Supplementary Fig. 1), comparable to that of hard spheres (*ϕ*_f_ = 49.4%)^58^.

We prepared the sample by loading the NIPA colloidal suspension into a glass channel and annealed it into fcc polycrystals with a few large domains in several days. In each domain of the crystal, the (111) face is parallel to the glass walls. The good refractive-index matching between particles and water enables us to see through all ~220 layers by using a bright-field Olympus BX63 microscope. By tuning the temperature, we precisely changed the volume fraction over a substantial range to melt and recrystallise the sample. We repeated this cycle several times to release possible stress in crystals.

We adjusted the ambient temperature *T*_ambient_ by uniformly heating ~1.8 cm^2^ of the sample with temperature controllers (Bioptechs) at a resolution of 0.1 °C. To avoid a temperature gradient along the *z* direction of the sample under uniform heating, two heating controllers (Bioptechs) were placed on both sides of the sample. One is on an oil immersion 100× objective and the other is on an Achromatic/Aplanatic condenser with oil between the top lens and glass sample cell for better heat conduction. The two heating systems were calibrated such that melting layers on GBs exhibit a uniform width in the *z* direction, which means that the temperature difference is less than the resolution of 0.1 °C throughout the sample cell.

Our experiments were conducted in an isothermal system (within a 1.0 °C temperature variation), in contrast to the isobaric systems commonly employed in simulations and atomic systems. However, the melting mechanism does not depend on the route by which the melting line is approached.

### Local heating technique

Besides the uniform heating by the heating controllers, to extract the melting behaviours of single GBs, we focused a beam of light emitted from a 100 W mercury lamp by the objective through the reflection mode to heat single GBs in the interior of the polycrystals, which causes an extra temperature increase *δT* ≈ 1.0 °C in the heated region. *δT* is equal to the difference of ambient temperatures at the melting point with and without local optical heating. A small amount (0.1% by volume) of nonfluorescent liquid dye (D980101 Chromatint jet black 1990 Chromatech Incorporated) was added to the sample to absorb the heating light. The dye seems to have a minor effect on the particle interaction in a short time after preparation^46,59^. The heating effect saturates in ~2 s after we turned on the light^42^. The size of the heating region and *δT* can be independently and continuously tuned by adjusting the field iris and aperture iris, respectively. Usually, *δT* is set to ~1.0 °C at the centre of the object plane, and the built-in fluorescence illuminators (Olympus BX3-RFAA) equipped with a Fly-eye-lens system promise an even and uniform heating effect. In addition, we placed two extra pieces of paraffin films in the light path so that the temperature variation is less than 0.1 °C (i.e., ~0.5% in volume fraction) in the 4*π*/3(100^2^ × 60) μm^3^ region of interest, which contains ~2.0 × 10^6^ particles. The region corresponding to a 0.1 °C decay in the *z* direction from the object plane is indicated by the width of the melted GB layers when we locally heated a central triple junction. The temperature at the centre was purposely set to a point that is higher than the melting temperature, such as *T*_m_ + 0.2 °C. Consequently, the widths of the layers within the region are larger than those measured under uniform heating at *T*_m_ + 0.1 °C (Fig. 2e). The profile of the temperature increase *δT* on the object plane (Fig. 1b) was further specified by measurement from a similar size glass channel containing NIPA suspension with an aqueous solution of fluorescein (0.03% by weight). The brightness of the fluorescent solution is proportional to the light intensity and heating effect^60^. The optical heating effect decays to zero at 60 μm outside the region of interest. The temperature is sufficiently uniform within the region of interest because we observed random nucleation on the GB interface. In most cases, we fixed *δT* and tuned *T*_ambient_. This local heating technique has been used to study homogeneous melting^42^ and nucleation during solid–solid transition^61^.

### Data acquirement and analysis

To capture the initial stage of heterogeneous nucleation on GBs in the assessment of incubation time (Fig. 3e), we manually scanned ±40 μm in the *z* direction in 3 s every 20 s. The rapid scanning does not affect the optical heating much. The 3-s uncertainty is much less than the shortest measured incubation time *τ* = 480 s. We only chose the nucleation that occurred on the object plane in the dynamics demonstrations (i.e., Supplementary Videos 2, 3 and 5), so the largest cross-section of the nucleus is always on the plane. Data were acquired after an equilibration time of 12 min each time *T*_ambient_ was changed by 0.1 °C. In all, 12 min is much longer than the characteristic time (*σ*^2^/*D*_0_ = 2.80 s). Raw images were captured by a charge-coupled device camera at 5–15 frames per second (fps), and the spheres’ positions were located by widely adopted routines written in Interactive Data Language^62^ for crystalline phase, but not for the liquid phase owing to their blurry images. For ease of observation, we selected flat GBs that have a length of two to three times the scale of the field of view and are vertical to the *x*−*y* plane. Therefore, all the GBs in the study are pure 〈111〉 tilt GBs. All the selected triple lines are also along the *z* direction.

With respect to the measurement of the critical nucleus in Fig. 1c, since the incubation time for spontaneous homogeneous nucleation is extremely long for very weak superheating (e.g., Δ*T* ≤ 0.3 °C and Δ*ϕ* ≲ 0.018), we directly burned a postcritical liquid nucleus inside a perfect crystalline domain by applying strong optical heating (*δT* ≈ 2.0 °C). When the produced liquid nucleus had grown to a desirable size, we changed the intensity of optical heating to normal settings. The critical nucleus was confirmed by adjusting *T*_ambient_ by ±0.1 °C so that the nearly stable spherical liquid nucleus has an equal probability of growing or shrinking. The r^*^ is estimated from the middle cross-section of the critical nucleus. The largest critical nucleus we measured is ~50 μm in diameter, observably smaller than the local heating region. Thus, Δ*ϕ* can be regarded as a constant during nucleation. The above method was also used to measure the critical nuclei on GBs (Fig. 3c) in conditions under which the incubation times are not measurable as indicated by the open symbols in Fig. 3g. Since the critical nucleus has been stabilised for a long time (~30 min), we did not observe significant deformation in the flat GB before each measurement. Any possible deformation in GB configuration that may appear during the melting-recrystallisation cycle of the GB^63^ has relaxed.

For raw images containing both solid and liquid phases, we smoothed the positions of particles at the solid–liquid interfaces (dashed lines in Figs. 2a, c, d and 3a), for which the 4-s time-averaged orientational order parameter^64^$\,{\langle | {\psi }_{6j}| \rangle }_{t}={\langle | \mathop{\sum }\nolimits_{k = 1}^{{Z}_{j}}{e}^{6i{\theta }_{jk}}| /{Z}_{j}\rangle }_{t} < \,0.6$, where *θ*_*jk*_ denotes the angle of the bond between reference particle *j* and its neighbour *k*. *Z*_*j*_ is the number of nearest neighbours identified from the Delaunay triangulation. Different criteria^47^ yield similar positions because of the sharp interface for hard-sphere colloidal systems^65,66^.

### Measurement of the pair potential

Pair potential *U*(*r*) (Supplementary Fig. 2) was extracted by analysing the radial distribution function *g*(*r*) (inset of Supplementary Fig. 2) with the Ornstein–Zernike integral equation of the liquid-structural theory^67^. Here, *g*(*r*) was measured in dilute fluids of the monolayer of spheres at an areal density of ~9.8%. Raw images were taken at 5 fps for 2 h. Image artefacts^68^ were corrected using the method described in ref. ^69^. Both the Percus–Yevick and hypernetted-chain approximation algorithms of the liquid-structure theory produce error bars smaller than 0.1 *k*_B_*T* of *U*(*r*) between samples. The measured *U*(*r*) is in close proximity to hard spheres, which is robust by the fact that the measured melting/freezing volume fractions (Fig. 1 and Supplementary Fig. 1) differ little from those for hard spheres.

## Supplementary information

Supplementary Information

Peer Review File

Description of Additional Supplementary Files

Supplementary Video 1

Supplementary Video 2

Supplementary Video 3

Supplementary Video 4

Supplementary Video 5

## Data Availability

The data for Figs. 1–3 in this study are provided in the Source Data files. All other relevant data are available from the corresponding author upon reasonable request. Source Data are provided with this paper.

## Source data

Source Data

## Code availability

The analysis codes that support the findings of this study are available from the corresponding author upon reasonable request.
