# Supplementary material for: Superheating of grain boundaries within bulk colloidal crystals
Source: Nat Commun. 2022 Mar 24;13:1599. doi: 10.1038/s41467-022-29254-z (PMC8948282; doi:10.1038/s41467-022-29254-z)
Supplement: Supplementary file 1 — Supplementary Information [file 41467_2022_29254_MOESM1_ESM.pdf]

Supplementary Information for “Superheating of grain boundaries within bulk colloidal crystals”

Xiuming Xiao, Lilin Wang, Zhijun Wang and Ziren Wang

This PDF file includes:

Supplementary Figures 1-6

Supplementary Notes 1-4

Supplementary References

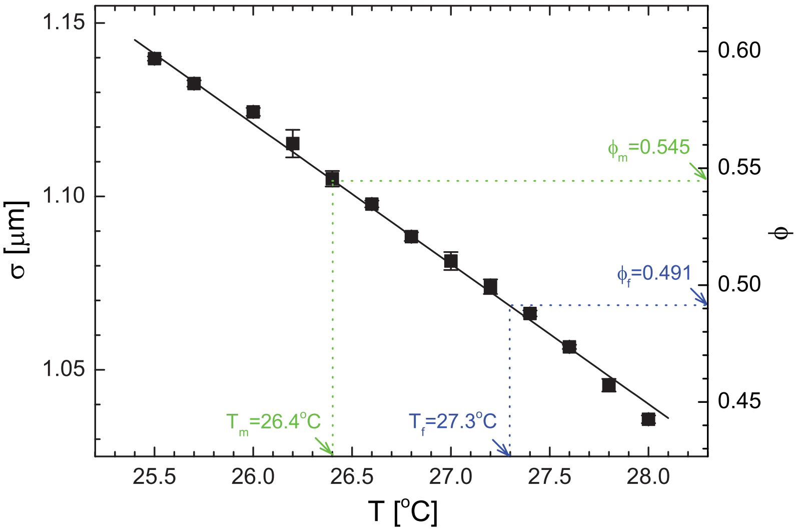

Supplementary Figure 1. Particle diameter variation with temperature and associated volume fraction ϕ. Error bars correspond to the standard deviation.

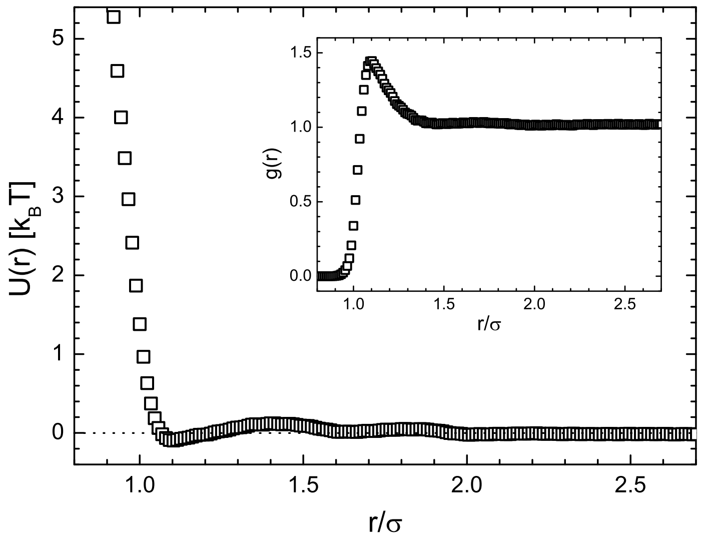

Supplementary Figure 2. Pair potential $U(r)$ extracted from the radial distribution function $g(r)$ (inset) at 26.4°C. $U(r)$ is very close to hard spheres and changes little within the temperature range of our experiments.

Supplementary Note 1. Measurement of the lindemann parameter

The dimensionless Lindemann parameter L, introduced in 1910 by Lindemann [1], has been widely used to quantify the average vibrating amplitude for crystalline particles. L is defined as the variance of the particle's displacement about the equilibrium lattice position, divided by the nearest-neighbor distance in crystals. The L is written as

$$L = \frac{1}{r_{\text{nn}}} \sqrt{\frac{1}{2}\frac{3}{2}\langle[\mathbf{r}(t \to \infty) - \mathbf{r}(0)]^2\rangle}, \tag{S1}$$

where r_{nn} is the nearest-neighbor distance in crystals which can be read from the first peak of the radial distribution function $g(r)$ (inset of Fig. 2), and $\langle[\mathbf{r}(t \to \infty) - \mathbf{r}(0)]^2\rangle$ is the asymptotic value of the two-dimensional (2D) MSD (inset 1 of Fig. 1c). $\langle\rangle$ denotes a statistical average over crystalline particles. The MSD asymptotically approaches a constant in 4 s because the particles are caged by the nearest neighbors. The factor 1/2 in Eq. S1 arises from the fact that the asymptotic constant corresponds to twice the variance of the particle's displacement from its equilibrium position [2, 3]. Since we monitored the dynamics on a 2D plane, the factor 3/2 in Eq. S1 is added to modify the 2D MSD into a 3D MSD under the assumption that particle fluctuations are isotropic in the three dimensions [2, 4]. The raw images were recorded at 15 fps.

Supplementary Note 2. Ratio between r^* and $\Delta\phi$ for homogeneous nucleation

During homogeneous nucleation, the free energy change for a spherical liquid nucleus of radius r is given by [5]

$$\Delta G_{\text{homo}}(r) = -\frac{4}{3}\pi r^3 \rho_l \Delta\mu + 4\pi r^2 \gamma_{sl} + E_{\text{strain}}, \tag{S2}$$

where $\Delta\mu$ is the chemical potential difference, ρ_l is the number density of the liquid phase, and γ_{sl} is the interfacial tension at the solid-liquid interface. The strain energy E_{strain} arises from the volume misfit between solids and liquids, which is proportional to the volume of the nucleus. In NIPA colloidal systems, E_{strain} is a negligible term [6], in agreement with the fact that we did not observe significant lattice distortions or cracks when nuclei grew. The critical radius $r^* = 2\gamma_{sl}/(\rho_l\Delta\mu)$, associated with a maximum free energy $\Delta G^*_{\text{homo}} = 16\pi\gamma_{sl}^3/[3(\rho_l\Delta\mu)^2]$. γ_{sl} is presumed as a constant in the vicinity of the melting point [7], and it has an average value $\gamma_{sl} \approx 0.60\ k_B T/\sigma^2$ according to previous numerical [8–11] and experimental [12] estimates. Therefore, to obtain the ratio between r^* and $\Delta\phi$, we calculated ρ_l and $\Delta\mu$ in terms of $\Delta\phi$, based on the framework of hard spheres.

For an isothermal process, the differential form of the chemical potential $\mathrm{d}\mu = \mathrm{v}\mathrm{d}p$, where v is the volume per particle and p is the pressure of the system. Therefore, $\Delta\mu$ can be written as

$$\begin{aligned}\Delta\mu \equiv \mu_s(\phi_s) - \mu_l(\phi_l) &= \int_{0.545}^{\phi_s} \mathrm{v}_s \frac{\mathrm{d}p_s}{\mathrm{d}\phi_s}\mathrm{d}\phi_s - \int_{0.491}^{\phi_l} \mathrm{v}_l \frac{\mathrm{d}p_l}{\mathrm{d}\phi_l}\mathrm{d}\phi_l \\ &= \int_{0.545}^{\phi_s} \frac{\mathrm{d}p_s}{\mathrm{d}\rho_s}\frac{\mathrm{d}\phi_s}{\phi_s} - \int_{0.491}^{\phi_l} \frac{\mathrm{d}p_l}{\mathrm{d}\rho_l}\frac{\mathrm{d}\phi_l}{\phi_l},\end{aligned} \tag{S3}$$

where $\phi = \pi\rho\sigma^3/6$ and the number density $\rho \equiv 1/\mathrm{v}$. Then we evaluated $\mathrm{d}p/\mathrm{d}\rho$ using the Kolafa-Labík-Malijevský equation of state (EOS) for hard-sphere liquids [13]

$$\begin{aligned}p_l/(\rho k_B T) =& 1 + 4x + 6x^2 + 2.3647684x^3 - 0.8698551x^4 \\ &+ 1.1062803x^5 - 1.095049x^6 + 0.637614x^7 \\ &- 0.2279397x^{10} + 0.1098948x^{14} - 0.00906797x^{22}\end{aligned} \tag{S4}$$

and the revised Speedy EOS for hard-sphere fcc crystals [14]

$$p_s/(\rho k_B T) = \frac{3}{1-z} - \frac{a(z-b)}{z-c}, \tag{S5}$$

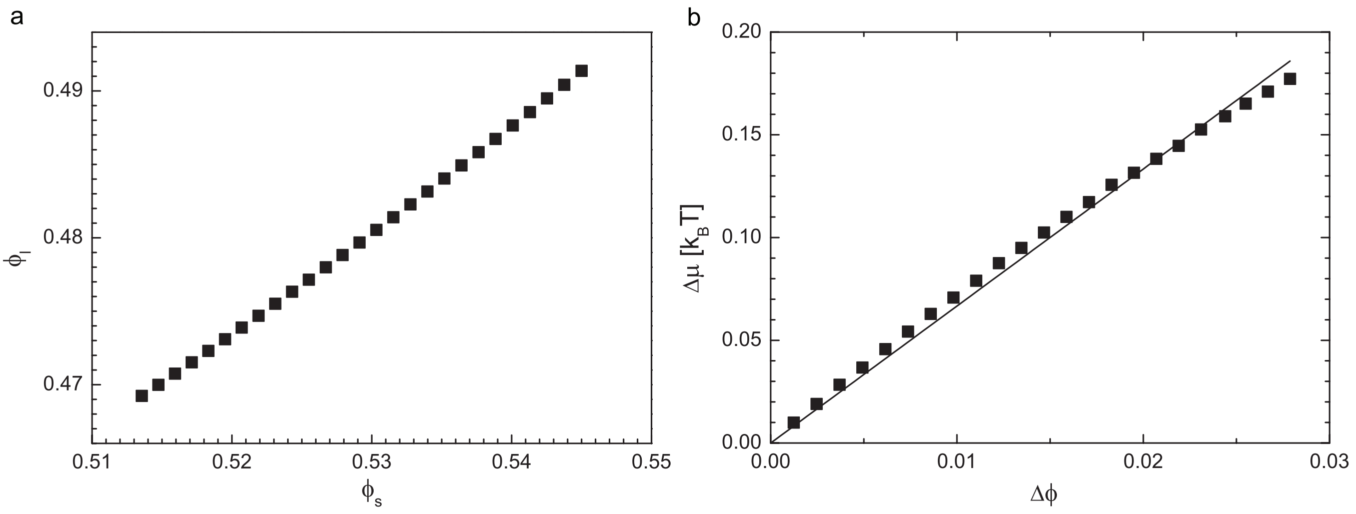

Supplementary Figure 3. (a) ϕ_s and the corresponding ϕ_l calculated on the basis of Eqs. S4 and S5 under equipressure condition. (b) The calculated $\Delta\mu$, which can be well fitted by $\Delta\mu/k_B T = 6.7\Delta\phi$ under weak superheating.

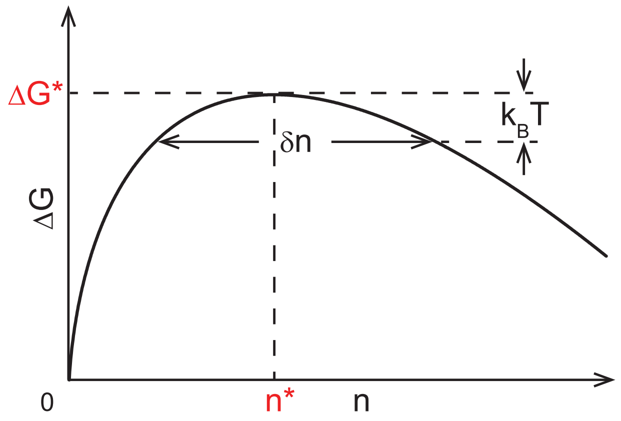

Supplementary Figure 4. The schematic of the free energy ΔG for a nucleus containing n particles, showing the width of the critical region δn.

where $x = \phi/(1-\phi)$, $z = \rho\sigma^3/\sqrt{2}$, $a = 0.620735$, $b = 0.708194$ and $c = 0.591663$. Eqs. S4 and S5 fit the numerical simulation result $p/(\rho k_B T)$ with an error of less than 4×10^{-5} when $\phi_l < 0.539$ [14] and less than 10^{-5} when $\phi_s > 0.497$ [13] respectively. Therefore, they can be used to accurately evaluate $\Delta\mu$ in our $0.520 < \phi_s < 0.545$ (ϕ_s is the ϕ denoted in the main text). Accordingly, ϕ_l is calculated from the equipressure condition $p_s(\phi_s) = p_l(\phi_l)$ (Fig. 3a) based on Eqs. S4 and S5. Figure 3b presents $\Delta\mu$ as a function of $\Delta\phi$, where $\Delta\mu$ is well fitted by $\Delta\mu/k_B T = 6.7\Delta\phi$ under weak superheating. Finally, we obtained $r^*/\sigma = 0.195/\Delta\phi$. This relationship has been adopted as a standard by which to evaluate our measurements in Fig. 1c.

Supplementary Note 3. Incubation time τ for nucleation on GBs

As sketched in Fig. 4, the incubation time τ is equal to the time required for the monomer to form a nucleus with a size $n^* - \delta n/2$ plus the subsequent time required for the nucleus to random walk across δn with a diffusivity ΓS_n [15]. Usually, the former time is negligible in comparison to the latter one. Therefore, τ is approximated as $\delta^2 n/(2\Gamma S_n)$, where Γ is the rate

of a successful jump for a particle crossing the nucleus interface and S_n is the number of particles adjacent to the solid-liquid interface when the nucleus size is n.

For heterogeneous nucleation on GBs, the nuclei are composed of two abutted spherical caps on either side of the GB face. Each cross-section displays a similar shape in the z scan (Movie 3). The free energy change is given as

$$\Delta G_{\mathrm{b}}(r) = -\frac{2}{3}\pi r^3 \rho_{\mathrm{l}} \Delta\mu (2+\cos\beta)(1-\cos\beta)^2 + 4\pi r^2 \gamma_{\mathrm{sl}}(1-\cos\beta) - \pi r^2 \sin^2\beta\gamma_{\mathrm{b}}, \tag{S6}$$

where r is the cap radius and β is the contact angle as defined in Fig. 3a. Combining with the relationship $\gamma_{\mathrm{b}}/(2\gamma_{\mathrm{sl}}) = \cos\beta$, we obtained the critical radius $r^* = 2\gamma_{\mathrm{sl}}/(\rho_{\mathrm{l}}\Delta\mu)$, identical to that for homogeneous nucleation. The energy barrier $\Delta G^*_{\mathrm{b}} = [(2+\cos\beta)(1-\cos\beta)^2/2]\Delta G^*_{\mathrm{homo}}$. GBs lower the energy barrier by a factor of $(2+\cos\beta)(1-\cos\beta)^2/2 \leq 1$, relative to that of homogeneous nucleation (inset of Fig. 3g). Substituting the measured β (Fig. 3b), we found that larger θ' leads to a greater reduction in the energy barrier at a given degree of superheating (Fig. 3g), as expected. Given that nucleus size $n = 2\pi r^3 \rho_{\mathrm{l}}(2+\cos\beta)(1-\cos\beta)^2/3$, we obtained the critical size $n^* = 16\pi\gamma_{\mathrm{sl}}^3(2+\cos\beta)(1-\cos\beta)^2/(3\Delta\mu^3\rho_{\mathrm{l}}^2)$. Accordingly, Eq. S6 is converted to be

$$\Delta G_{\mathrm{b}}(n) = -n\Delta\mu + \gamma_{\mathrm{sl}}[18\pi(2+\cos\beta)]^{1/3}[n(1-\cos\beta)/\rho_{\mathrm{l}}]^{2/3}. \tag{S7}$$

Next, the explicit expression of δn is derived by expanding the free energy $\Delta G(n)$ at n^*

$$\Delta G_{\mathrm{b}}(n) = \Delta G_{\mathrm{b}}(n^*) + \frac{(n-n^*)^2}{2}\left.\frac{\partial^2 \Delta G_{\mathrm{b}}(n)}{\partial n^2}\right|_{n=n^*} = \Delta G_{\mathrm{b}}(n^*) - \frac{(n-n^*)^2}{2}\frac{\rho_{\mathrm{l}}^2\Delta\mu^4}{16\pi\gamma_{\mathrm{sl}}^3(2+\cos\beta)(1-\cos\beta)^2}. \tag{S8}$$

As shown in Fig. 4, we have $\Delta G_{\mathrm{b}}(n^* - \delta n/2) = \Delta G_{\mathrm{b}}(n^*) - k_{\mathrm{B}}T$, then $\delta^2 n = 128\pi k_{\mathrm{B}} T\gamma_{\mathrm{sl}}^3(2+\cos\beta)(1-\cos\beta)^2/(\rho_{\mathrm{l}}^2\Delta\mu^4)$. Meanwhile, S_n can be replaced as $S_{n^*} = 4\pi l_{\mathrm{a}}\rho_{\mathrm{l}} r^{*2}(1-\cos\beta)$, where $l_{\mathrm{a}} \approx \sigma\phi_{\mathrm{l}}^{-1/3}$ is the particle spacing. Eventually, we obtained

$$\tau = \frac{4k_{\mathrm{B}}T\gamma_{\mathrm{sl}}}{\Delta\mu^2 l_{\mathrm{a}}\rho_{\mathrm{l}}\Gamma}(2+\cos\beta)(1-\cos\beta). \tag{S9}$$

Under weak superheating, τ is further reduced to $0.049(2+\cos\beta)(1-\cos\beta)\Delta\phi^{-2}/\Gamma$. The factor $(1-\cos\beta)(2+\cos\beta)$ increases by 50% as the degree of superheating rises, thus $\tau \propto \Delta\phi^{-2}$.

Supplementary Note 4. Measurement of γ_{b} from GB fluctuations

We conducted a supplemental experiment to measure γ_{b} by the approach of quantifying the thermal fluctuations of unperturbed GBs [16]. The sample is composed of thin-film NIPA colloidal crystals sandwiched between two parallel glass plates with an optimal thickness of ~7 μm (7 layers). We specially prepared the sample in thin-film crystals instead of three-dimensional crystals, to ensure that the GB has a ribbon-like shape with a uniform configuration along the z direction under fluctuations. Thus, the data acquired on the object plane can represent the motion of the whole GB interface that is vertical to the $x-y$ plane. Simultaneously, we assume that for thin-film crystals at this thickness, the triple line energy at the intersections of the grain boundaries with the glass plates has such minor effect that the measured γ_{b} can replace the true value. Before the measurement,

we stored the sample in an incubator for one week at temperature $T_\mathrm{m} - 0.2$°C to ensure a stable GB. Raw images were taken at 1 fps for ~2.0 hours at the same temperature under uniform heating. The temperature is set to be slightly below the melting temperature to avoid any momentary blur in the image, thereby improving accuracy while locating GB positions.

We determined the position of a GB using the methodology described in Ref. [16]. First, a local orientation parameter $\varphi_j = \sum_{k=1}^{Z_j} \theta_{jk}/Z_j$ is assigned to each particle. θ_{jk} denotes the angle of the bond between reference particle j and its neighbor k, with $-\pi < \theta_{jk} \leq \pi$. Z_j is the number of nearest neighbors identified from the Delaunay triangulation. Next, for a vertical GB shown in Fig. 5, the image is divided into horizontal bins at a size of the nearest-neighbor distance r_nn which can be read from the first peak of the radial distribution function $g(r)$ (inset of Fig. 2). Each bin offers an interfacial point (x, y) that indicates the position at which the lattice orientation switches. Consequently, the position of a GB can be described in terms of separated interfacial points (orange circles in Fig. 5). For each bin, x is given by the following tangent-hyperbolic fit

$$\varphi_j(x_j) = A \tanh(Bx_j + C) + D, \tag{S10}$$

where A, B, C and D are fitting parameters. x_j represents the position of the center of each particle within the bin. x is the one corresponding to the inflection point of the fitting function and y is taken as the position of the center of the bin.

The length of a given GB is calculated as the sum of distances between adjacent interfacial points. The probability of forming a GB with a length s under thermal fluctuations is proportional to the Boltzmann factor. That is

$$P(s) \propto \Omega(s)\mathrm{e}^{-\gamma_\mathrm{b} sh/k_\mathrm{B}T}, \tag{S11}$$

where $h = 6.3\ \sigma$ is the thickness of the thin-film crystals. $\Omega(s)$ represents the number of possible configurations, which is hard

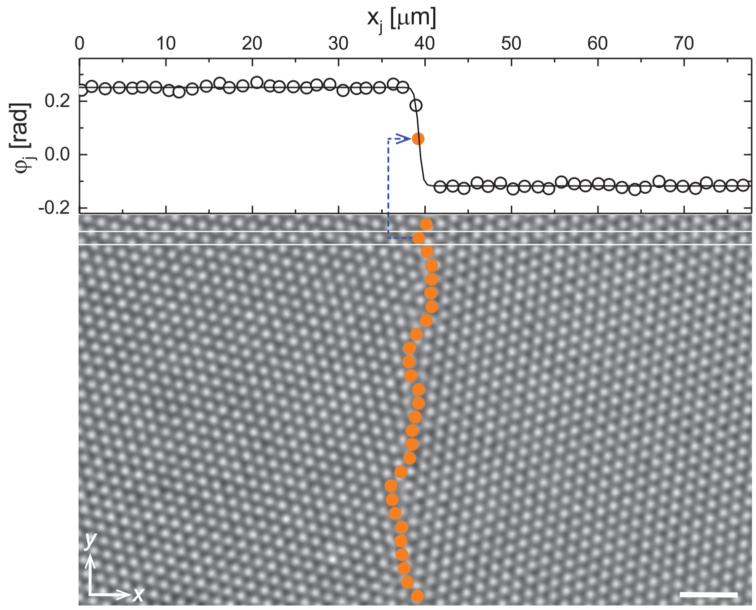

Supplementary Figure 5. Determination of the position of a GB with $\theta = 22°$. The vertical GB is divided into horizontal bins at the size of the nearest-neighbour distance r_nn. For the specified bin shown in two parallel white lines, an interfacial point indicated in an orange circle is given by the hyperbolic tangent fit of a local orientation parameter φ_j as a function of the particle's position x_j within the bin. Therefore, the GB position is expressed in terms of individual interfacial points. Scale bar: 5 μm.

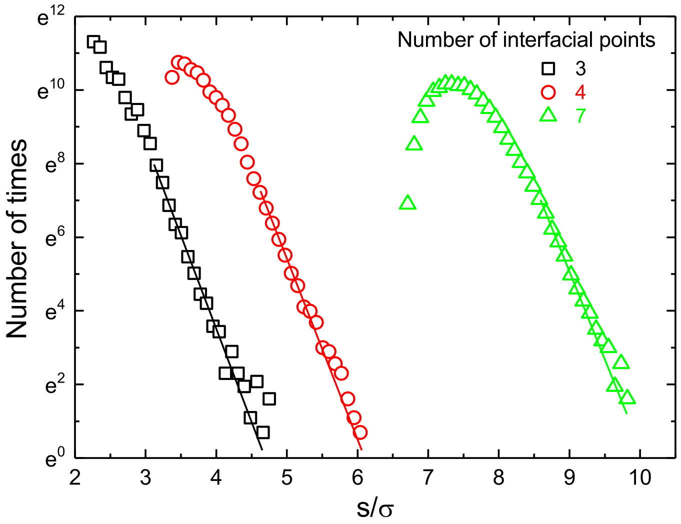

Supplementary Figure 6. Histograms of GB length s consisting of three, four and seven interfacial points, fitted by Eq. S11 at larger s. The fitted γ_b are 0.80 $k_B T/\sigma^2$, 0.77 $k_B T/\sigma^2$ and 0.76 $k_B T/\sigma^2$ respectively.

to quantify. However, for larger s, the Boltzmann factor should predominate over $\Omega(s)$, responsible for the frequency change. Consequently, $\Omega(s)$ can be treated as a constant.

In Fig. 6, we plotted the length histograms of three fluctuated GBs that consist of three, four and seven neighbouring interfacial points respectively. The three curves yield an average value γ_b = 0.78 $k_B T/\sigma^2$ by fitting Eq. S11.

Supplementary References

---

[1] Lindemann, F. A. The calculation of molecular vibration frequency. *Z. Phys.* **11**, 609–612 (1910).

[2] Bongers, J. & Versmold, H. Microscopic investigations of the single particle dynamics in colloidal crystals. *J. Chem. Phys.* **104**, 1519–1523 (1996).

[3] Ohshima, Y. N. & Nishio, I. Colloidal crystal: bead-spring lattice immersed in viscous media. *J. Chem. Phys.* **114**, 8649–8658 (2001).

[4] Alsayed, A. M., Islam, M. F., Zhang, J., Collings, P. J. & Yodh, A. G. Premelting at defects within bulk colloidal crystals. *Science* **309**, 1207–1210 (2005).

[5] Porter, D. A., Easterling, K. E. & Sherif, M. Y. *Phase Transformations in Metals and Alloys* (CRC press, 2009).

[6] Wang, Z., Wang, F., Peng, Y., Zheng, Z. & Han, Y. Imaging the homogeneous nucleation during the melting of superheated colloidal crystals. *Science* **338**, 87–90 (2012).

[7] Frolov, T. & Mishin, Y. Liquid nucleation at superheated grain boundaries. *Phys. Rev. Lett.* **106**, 155702 (2011).

[8] Auer, S. & Frenkel, D. Prediction of absolute crystal-nucleation rate in hard-sphere colloids. *Nature* **409**, 1020–1023 (2001).

[9] Davidchack, R. L. Hard spheres revisited: Accurate calculation of the solid-liquid interfacial free energy. *J. Chem. Phys.* **133**, 234701 (2010).

[10] Härtel, A. *et al.* Tension and stiffness of the hard sphere crystal-fluid interface. *Phys. Rev. Lett.* **108**, 226101 (2012).

[11] Bültmann, M. & Schilling, T. Computation of the solid-liquid interfacial free energy in hard spheres by means of thermodynamic integration. *Phys. Rev. E* **102**, 042123 (2020).

[12] Nguyen, V. D., Dang, M. T., Weber, B., Hu, Z. & Schall, P. Visualizing the structural solid-liquid transition at colloidal crystal/fluid interfaces. *Adv. Mater.* **23**, 2716–2720 (2011).

[13] Kolafa, J., Labík, S. & Malijevskỳ, A. Accurate equation of state of the hard sphere fluid in stable and metastable regions. *Phys. Chem. Chem. Phys.* **6**, 2335–2340 (2004).

[14] Bannerman, M. N., Lue, L. & Woodcock, L. V. Thermodynamic pressures for hard spheres and closed-virial equation-of-state. *J. Chem. Phys.* **132**, 084507 (2010).

[15] Russell, K. C. Grain boundary nucleation kinetics. *Acta Metall.* **17**, 1123–1131 (1969).

[16] Skinner, T. O. E., Aarts, D. G. A. L. & Dullens, R. P. A. Grain-boundary fluctuations in two-dimensional colloidal crystals. *Phys. Rev. Lett.* **105**, 168301 (2010).
