# Supplementary material for: Superheating of grain boundaries within bulk colloidal crystals
Source: Nat Commun. 2022 Mar 24;13:1599. doi: 10.1038/s41467-022-29254-z (PMC8948282; doi:10.1038/s41467-022-29254-z)
Supplement: Supplementary file 3 — Description of Additional Supplementary Files [file 41467_2022_29254_MOESM3_ESM.pdf]

# Description of Additional Supplementary Files for "Superheating of grain boundaries within bulk colloidal crystals"

Xiuming Xiao, Lilin Wang, Zhijun Wang and Ziren Wang

File Name: Supplementary Video 1

Description: Melting process at a typical triple junction under uniform heating. The temperature increases from $T_{\rm m} - 0.2$°C to $T_{\rm m} + 0.2$°C with a step size of 0.1°C (60 × real time for each temperature).

File Name: Supplementary Video 2

Description: Nucleation on a GB ($\theta = 39°$) at $\Delta T = 0.2$°C (330 × real time). The largest cross-section of the nucleus is on the object plane.

File Name: Supplementary Video 3

Description: A critical nucleus on a GB ($\theta = 27°$) at $\Delta T = 0.2$°C scanned in the $z$ direction (2 × real time). The largest cross-section of the critical nucleus is selected as Fig. 3a of the main text.

File Name: Supplementary Video 4

Description: Melting of a GB ($\theta = 33°$) via direct widening at the superheat limit $\Delta\phi_{\rm limit} = 0.054$ or $\Delta T = 0.9$°C (2 × real time).

File Name: Supplementary Video 5

Description: Nucleation on a single dislocation at $\Delta T = 1.0$°C (115 × real time). The left side is the raw image, and the right side is the corresponding coloured Voronoi diagram for better visualization.
